# Psychometric Properties of the Apathy Scale in Advanced Parkinson's Disease

**DOI:** 10.1155/2019/1965394

**Published:** 2019-03-28

**Authors:** John B. Wetmore, José Matías Arbelo, María José Catalán, Francesc Valldeoriola, Carmen Rodriguez-Blazquez, Pablo Martinez-Martin

**Affiliations:** ^1^National Center of Epidemiology, Carlos III Institute of Health, Madrid 28029, Spain; ^2^Department of Epidemiology and Biostatistics, Graduate School of Public Health and Health Policy, City University of New York, New York, NY 10027, USA; ^3^Movement Disorders Unit, Hospital Universitario Insular de Gran Canaria, Las Palmas de Gran Canaria 35016, Spain; ^4^Department of Neurology, Hospital Clínico San Carlos, Madrid 28040, Spain; ^5^Department of Neurology, Hospital Clínico de Barcelona, Barcelona 08036, Spain; ^6^Center for Networked Biomedical Research in Neurodegenerative Diseases (CIBERNED), Madrid 28031, Spain

## Abstract

**Objectives:**

To assess the psychometric attributes of the Apathy Scale- (AS-) Spanish version in patients with advanced Parkinson's disease (APD).

**Materials and Methods:**

Over 6 months, 61 patients participated in a clinical study of levodopa-carbidopa intestinal gel (LCIG) and were evaluated using the AS and other clinical tools. Various psychometric attributes of the AS were assessed.

**Results:**

Patients (60.7% men) were aged 68.02 ± 7.43 years, with 12.57 ± 5.97 years from PD diagnosis. Median HY of patients in “on state” was 2 (range, 1–4), and mean levodopa equivalent daily dose was 1455.98 ± 456.00 mg. Overall, the parameters of feasibility/acceptability were satisfactory, except for a moderate-to-high floor effect in AS items but not in its total score (both 3.3%). Cronbach's alpha was 0.78, while item homogeneity coefficient was 0.21. Almost all items (11/14) reached acceptable item-total corrected correlations (*r*_S_ = 0.16–0.50). AS total score was moderately correlated with Beck Depression Inventory (0.34) and with Non-Motor Symptoms Scale domains 2 (sleep/fatigue, 0.35), 3 (mood/apathy, 0.56), and 5 (attention/memory, 0.41). There were no significant differences between AS total scores by established groups of sex, time from diagnosis, HY, and Clinical Global Impression-Severity Scale. Following LCIG treatment, there was no significant change in the AS total score. The relative change was 5.56%, the standard error of the difference was 4.17, and Cohen's *d* effect was 0.10.

**Conclusions:**

The AS showed satisfactory feasibility, acceptability, scaling assumptions, internal consistency, and convergent validity. Responsiveness parameters were poor, probably due to the characteristics of the clinical study from which these data came. This trial is registered with NCT02289729.

## 1. Introduction

Apathy, a syndrome defined by an overall lack of interest and motivation in emotional, cognitive, and goal-directed behavior, [1] is a nonmotor symptom frequently associated with various medical disorders, including Parkinson's disease (PD). Its prevalence in healthy elderly populations has been proposed to be up to 20%, but it reaches around 40% (range, 34.6%–45.0%) in those with Parkinson's disease (PD) [2]. Findings suggest that apathy in patients with PD may be directly related to the physiological changes of PD rather than psychological reactions or adaptation to illness. It has been hypothesized that apathy in PD may be caused by dopamine deficiency in the limbic areas of the brain although its pathophysiology is probably complex and heterogeneous [1, 2].

Apathy has been shown to reduce goal-directed behaviors and emotional reactivity, causing less adherence to treatments, faster cognitive decline, lower quality of life (QoL), functional impairment, and increased caregiver burden [3, 4].

To accurately assess this burdensome nonmotor symptom in PD, the Apathy Scale (AS) was created [5]. The AS is a 14-question inventory that evaluates the domains of apathy related to cognition, motivation, and interest. It has shown satisfactory psychometric properties in PD and has been recommended for screening apathy and evaluating its severity in this context [6]; however, it has not yet been validated for use in either the advanced PD (APD) population or in the Spanish population. Therefore, the objective of this study was to assess the psychometric attributes of the AS-Spanish version in a sample of Spanish patients with APD.

## 2. Materials and Methods

### 2.1. Design

Data for the present study are derived from the ADEQUA study (this trial is registered with NCT02289729), an observational, single-arm, postmarketing, nationwide, multicenter clinical study that was carried out in patients with APD with an indication for levodopa-carbidopa intestinal gel (LCIG) [7].

### 2.2. Patients

Inclusion criteria were as follows: (1) APD designation by a neurologist; (2) indication for LCIG according to the drug fact sheet; (3) age ≥18 years; and (4) signed informed consent. Patients who experienced severe motor fluctuations and dyskinesia were considered to have APD if conventional treatment options could not satisfactorily manage these complications.

Exclusion criteria were as follows: (1) absence of any inclusion criteria; (2) cognitive deterioration that could impede the accurate completion of questionnaires, confirmed by a score of <26 points on the Mini-Mental State Examination; and (3) contraindication to LCIG, according to the drug fact sheet of the product.

Considering the objectives of the ADEQUA study, it was determined that a sample size of 60 patients would be necessary. The sample was recruited from November 2014 to April 2016.

### 2.3. Ethical Issues

The study was conducted in accordance with the Declaration of Helsinki and with standard operating procedures that guaranteed compliance with Good Clinical Practice, as described in the ICH guidelines. The study was evaluated by the Spanish Agency of Medicines and Medical Devices and was approved by the Spanish autonomous communities and the ethics committees of the participant hospitals. Patients had to be ≥18 years and must have provided informed consent to participate in the study.

### 2.4. Assessments

Information regarding age, sex, ethnicity, civil status, education, employment, time from PD diagnosis, and current treatment was collected.

The AS is a 14-question inventory that is useful in screening the presence of apathy and determining its severity over the past 4 weeks. Each question is scored from 0 to 3, with the total score (range, 0–42) calculated by summing the scores of every item. Questions 1 through 8 are scored on a scale from 3 (“not at all”) to 0 (“a lot”), whereas questions 9 through 14 are scored on a scale from 0 (“not at all”) to 3 (“a lot”). A score of 14 or higher indicates the presence of apathy in a patient [5]. In addition to the AS, patients completed the following assessments during this study:

1. Unified Parkinson's Disease Rating Scale (UPDRS) [8], a 4-domain, 42-item evaluative instrument specific to PD. Domains III (motor examination), where scores range from 0 to 108, and IV (complications of therapy in the past week), where scores range from 0 to 23, were used in this study.
2. Schwab and England Scale (S&E), a common scale that assesses the capacity to perform activities of daily living in PD patients in 11 stages from 0% (completely dependent with vegetative dysfunction, bedridden) to 100% (completely independent, essentially normal). The version included in the UPDRS was used [8].
3. Hoehn and Yahr (HY), a classification system that measures the stages of PD from 1 to 5, with 1 indicating unilateral PD manifestation and 5 indicating the most severe disease [8].
4. Non-Motor Symptoms Scale (NMSS) [9], a scale consisting of 30 items that quantitatively evaluates the burden of nonmotor symptoms in patients with PD. It takes into account both the severity and frequency of these symptoms, which are separated into 9 domains: cardiovascular, sleep/fatigue, mood/apathy, perceptual problems/hallucinations, attention/memory, gastrointestinal tract, urinary function, sexual function, and miscellaneous.
5. Clinical Global Impression-Severity (CGI-S) Scale [10], a 7-point scale in which clinicians rate the severity of a patient's disease at the time of assessment. The ratings range from 1 (“normal, not at all ill”) to 7 (among the most extremely ill patients”).
6. Beck Anxiety Inventory (BAI) [11], which is composed of 21 items scored between 0 and 3. Total scores are calculated by summing the items, with higher scores indicating more severe anxiety symptoms. This questionnaire determines the severity of anxiety in patients over the past week.
7. Beck Depression Inventory II (BDI-II) [12], which is composed of 21 items that range from 0 to 3 and that are summed to create a total score, with higher scores indicating more severe depressive symptoms. This assessment measures the severity of depression over the past 3 weeks.
8. Parkinson's Fatigue Scale (PFS-16) [13], a 16-item scale in which each item is scored between 1 (“strongly disagree”) and 5 (“strongly agree”). Scores range from 16 to 80, with higher scores indicating higher fatigue severity.
9. Parkinson's Disease Questionnaire-39 (PDQ-39) [14], a 39-item, patient-completed questionnaire that assesses QoL in patients with PD. These items are grouped into 8 dimensions, which include mobility, activities of daily living, emotional well-being, stigma, social support, cognitive deterioration, communication, and bodily discomfort.
10. Zarit Burden Interview (ZBI) [15], an instrument designed to assess the burden experienced by caregivers of dependent persons. It consists of 22 items that range from 0 (“never”) to 4 (“nearly always”) and that, when summed, represent the level of caregiver burden.

Patient assessment was performed in “ON” state. Levodopa equivalent daily dose (LEDD) was also calculated for each patient according to Tomlinson et al. [16].

### 2.5. Data Analysis

The data were anonymized by each center and were sent to the National Center of Epidemiology, Carlos III Institute of Health, Madrid, Spain.

As shown by the Shapiro–Francia test, the data distribution of the main variables in the study was not normal; therefore, nonparametric statistics were utilized. To calculate the AS total score, item scores were summed following the rules of the scale. For demographic data, *χ*^2^ and Mann–Whitney *U* tests were conducted to determine whether significant differences were present between groups.

Data quality and acceptability were calculated for each item of the AS, as well as for the total score, by determining the percentage of missing responses, mean, median, standard deviation, skewness, minimum and maximum values, and floor and ceiling effects. Concerning data quality, missing items should have been <5% of the data set. Similarly, data acceptability was considered satisfactory when all possible scores were observed, the mean and median were close (difference <10% of maximum possible score), floor and ceiling effects were <15%, and skewness values ranged from −1 to 1 [17]. Internal consistency was assessed using Cronbach's alpha (standard, >0.70) [17], item-total corrected correlation (*r* ≥ 0.30 was deemed acceptable), and item homogeneity coefficient (standard value, ≥0.15) [18].

Hypotheses testing was carried out using both convergent and known-groups validity. Spearman's rank correlation coefficients were utilized to analyze the relationship between AS total score and both the domains and total scores of the other assessments completed in this study. Convergent validity was considered high for correlation coefficients ≥0.60 and moderate for correlations coefficients between 0.30 and 0.59 [19]. Furthermore, Mann–Whitney and Kruskal–Wallis tests were used to determine whether significant differences existed in the AS total score between groups based on age, sex, time from diagnosis, HY, and CGI-S (known-groups validity). Based on Fayers and Machin, these differences were considered significant if the magnitude of the difference was evident (e.g., ≥15% of the maximum total score) and *p* < 0.05 [20].

A longitudinal analysis was conducted using the data collected at follow-up, which took place 6 (±0.5) months later. AS responsiveness was determined by the magnitude of the difference between baseline and follow-up, relative change, standard error of the difference ($S_{\text{diff}}=\sqrt{{\text{SEM}}_{1}^{2}+{\text{SEM}}_{2}^{2}}$) [21], and effect size for paired data between applications. Effect size was considered small (0.20–0.49), moderate (0.50–0.79), or large (≥80). In addition, correlation coefficients were obtained to show the association between the change in the AS total score and the scores of the other measures in the study. The data analysis was conducted using IBM SPSS (Version 24; IBM Corp, Armonk, NY).

## 3. Results

At baseline, our sample included 61 patients and was composed of 37 (60.7%) men, with a mean age of 68.02 ± 7.43 (range, 50–81) years. Patients were diagnosed with PD 12.57 ± 5.97 (range, 4–37) years ago. Most patients were married/partnered (77.05%) and retired (78.7%), and their predominant level of education was basic or less (77.0%). The median HY stage of patients in the “on state” was 2 (interquartile range, 1-2), with 44.26% of patients in stage 1, 42.62% in stage 2, 11.48% in stage 3, and 1.64% in stage 4. Concerning the CGI-S (median, 3; range, 1–4), most patients had a score of 3 (75.41%) or 4 (14.75%); however, scores of 1 (1.64%) and 2 (8.20%) were also represented. One patient did not complete the AS and, therefore, the final sample was of 60 patients.  Table 1 shows the descriptive statistics for the applied measures.

For AS items, there was only one missing datum (0.12%), which was imputed by the mean of the individual's observed values. Concerning item-related acceptability, all possible scores were observed in the sample, except in items 2 and 4. The standard deviation of items ranged from 0.62 to 1.06, and they showed a moderate-to-high floor effect (Table 2). Most item scores remained in the accepted range of skewness (−1 to +1) or were marginally outside (up to an excess of |0.81| points). The mean AS total score was 11.55 ± 6.49 (median, 10; range, 1–24), with negligible floor and ceiling effects (both, 3.3%) and a skewness value of 0.32 (Table 2). The cutoff of ≥14 points indicative of apathy [5] was reached by 36.7% of the sample.

Regarding internal consistency, Cronbach's alpha was 0.78, while item homogeneity coefficient was 0.21. As shown in Table 3, almost all items (11/14) reached item-total corrected correlations above the criterion 0.30, except for items 3 (0.16), 4 (0.22), and 13 (0.21).

The AS total score was moderately correlated with age (*r*_S_ = 0.32); NMSS items 7 (loss of interest in the patient's surroundings, 0.38) and 8 (loss of interest in doing things or lack of motivation to start new activities, 0.56); NMSS domains 2 (sleep/fatigue, 0.35), 3 (mood/apathy, 0.56), and 5 (attention/memory, 0.41); NMSS total score (0.49); and BDI-II (0.34). However, its associations with the rest of the PD-related measures were weak or negligible. Moreover, there were no significant differences between AS total scores by established groups of sex, time from diagnosis (<10 years, 10–15 years, >15 years, based on percentiles and tabulation), HY, and CGI-S.

In addition, longitudinal analysis of 53 patients who completed the study showed significant improvements in the conditions assessed by the quantitative instruments utilized in this study between baseline and follow-up, except for the AS and ZBI (Table 4). The relative change between applications for the AS was 5.56%, the standard error of the difference was 4.17, and Cohen's *d* effect was 0.10. Correlations between the change in AS and the change in other measures were moderate for NMSS (*r*_S_=0.56) and for PDQ-39 and UPDRS IV (both, *r*_S_=0.31), but they were weak for the others (*r*_S_=|0.01 – 0.21|).

## 4. Discussion

This is the first attempt to validate the Spanish version of the AS in patients with APD. The original English version, which is an adaptation of the Apathy Evaluation Scale, acceptably represents the cognitive, behavioral, and affective domains of the construct of apathy [22, 23].

The present study showed satisfactory AS data quality (99.9% of data available) and scaling assumptions, considering the range of item score distributions as a whole. A moderate-to-high floor effect was present for most items, due to the absence of the corresponding symptom in that proportion of patients. Although no one had an AS total score of 0, only 36.7% of patients were diagnosed with apathy by the scale (Table 2). Floor and ceiling effects were slight for the total score, which showed skewness in the standard range. These results indicate appropriate feasibility and acceptability of the scale, yet they could not be compared with other studies, as this analysis has not been previously conducted to the best of our knowledge.

The internal consistency of the AS was satisfactory as a whole, with Cronbach's alpha higher than the threshold value and close to values from previous studies in regular and early PD patients (alpha, 0.69–0.83) [5, 23, 24]. Notably, item 3 showed the lowest item-total correlation (*r*_S_ = 0.16, Table 3), a finding in line with previous studies that showed low interitem correlation or effect indicator for this item [23, 25].

Our results showed that there were moderate associations between AS total score and the apathy-specific questions of the NMSS, items 7 and 8. These apathy-specific questions inquire about the loss of interest in the patient's surroundings and loss of interest in doing things or the lack of motivation to start new activities. As these are aspects characteristic to apathy, it follows that the AS total score would be significantly correlated with these items although there are clear differences among these assessments in their structure and content. Overall, a moderate correlation was found between AS and NMSS total score. This association could be explained by the inclusion of NMSS domains specific to sleep/fatigue (domain 2), mood/apathy (domain 3), and attention/memory (domain 5) and disorders that have been shown to be associated with apathy [1, 26, 27].

However, the AS showed weak-to-moderate correlations with the BDI-II, PFS-16, and NMSS domain 2 (sleep/fatigue), suggesting a relatively loose relationship between apathy, depression, and fatigue in our sample. Although apathy is often combined with depression and fatigue, they are separate disturbances [24, 28].

In PD, moderate or high associations have been observed between the AS, including a reduced 11-item version, and depression measures [24, 25, 29, 30]. Nonetheless, a close correlation between apathy and depression is not universally observed [23, 26], and other studies had findings like ours regarding depression using different scales [31].

Similarly, an overlap between apathy and fatigue has been recognized although they are distinct disorders [32, 33]. A significant association between fatigue and apathy or specific apathy-related domains was observed in several studies [31, 34–36] but not in others [37]. The differences with the present study related to the sample (patients with APD) and the applied measures (the AS and PFS-16) make it difficult to compare the results. A similar situation occurs with the combination of sleep disorder and fatigue, with studies using different sample composition and measures [31, 36].

Findings from other studies do, however, confirm lower cognitive functioning in reference to attention and memory in PD, with higher levels of apathy in patients with cognitive impairment [38, 39]. Starkstein et al. demonstrated that patients with PD diagnosed with apathy according to the AS performed worse on time-dependent cognitive tasks than normal participants [5].

Due to the deficits in attention, memory, and concentration found in apathetic patients with PD, it had previously been proposed that apathy may be related to bradyphrenia and, thus, an alteration in catecholaminergic metabolism [5]. However, more recent studies have suggested that the presence of Lewy body and/or Alzheimer's pathology in these patients may lead to errors in memory encoding and retrieval [38]. Additional research is needed to definitively establish the relationship between apathy, depression, and cognitive impairment [40].

Previous research has also suggested that apathy is related to QoL [29], although the correlation between AS and PDQ-39 in that particular study was substantially higher than ours (*r*_S_ = 0.51 vs. 0.09), despite similar correlations between AS and UPDRS III, UPDRS IV, patient age, time from PD diagnosis, and LEDD in both studies. We can only explain this discrepancy due to the difference between samples: consecutive patients in the study by Oguru et al. [29] and patients with APD in ours.

Furthermore, the AS total score did not differ significantly between the known groups tested. This would suggest that apathy is not directly related to sex, time since PD diagnosis, HY stage, and illness severity levels (CGI-S). Although the presence of apathy has been associated with shorter time since PD diagnosis, earlier HY stages, and lower LEDD, these results were again observed in samples with patients in the earlier stages of PD [41]. Cognitive performance is also linked with level of education. In this study, the majority of participants had only a basic level of education that may have an influence in the apathy score.

Contrary to our findings in which the AS total score did not differ significantly following LCIG treatment, previous research has shown that levodopa and dopamine agonists may lessen the burden of apathy in patients with PD, as a dopaminergic deficit could contribute to the development of apathy; however, it is important to note that the pathophysiology of apathy is multifactorial in nature [1, 28]. If LCIG was an efficacious treatment for apathy, our longitudinal analysis showed poor responsiveness for the AS, with a standard error of the difference clearly higher than the mean difference between baseline and follow-up, a negligible effect size, small relative change, and weak or moderate correlations with changes in other clinical variables.

The primary limitation of our study is that it was not designed as a scale validation study but rather as a clinical study. Therefore, the study was limited by the lack of a gold standard to accurately assess the presence or absence of apathy in our patients with APD and to conduct a sensitivity and specificity analysis. Starkstein and colleagues reported a sensitivity of 66% and a specificity of 100% in their original sample using neurologist clinical impressions as their gold standard [5]; future research warrants a sound evaluation of this measure [34]. Using a Spanish version of the scale, our paper presents the first validation of the AS in patients with APD and a complementary assessment of this important clinical tool.

## 5. Conclusions

The AS as a whole showed satisfactory feasibility, acceptability, scaling assumptions, internal consistency, and convergent validity. Responsiveness parameters were poor, but data came from a clinical study of patients with APD with a nonspecific therapy for treating apathy, making it difficult to judge this psychometric attribute.

## Figures and Tables

**Table 1:** Descriptive statistics of other measures in the study.

Measures	Mean	SD	Min	Max
Beck Anxiety Inventory	19.77	9.24	0	41
Beck Depression Inventory-II	18.11	9.34	1	46
Schwab and England Scale (“on state”)	69.84	23.77	10	100
Non-Motor Symptoms Scale-total score	83.05	32.07	29	167
Cardiovascular (including falls)	2.41	3.53	0	14
Sleep/fatigue	16.87	7.63	0	40
Mood/cognition	15.69	13.93	0	56
Perceptual problems/hallucinations	3.08	5.53	0	25
Attention/memory	5.80	6.11	0	24
Gastrointestinal tract	7.44	6.23	0	24
Urinary	12.44	9.59	0	36
Sexual function	7.80	8.16	0	24
Miscellaneous	11.51	8.68	0	36
PDQ-39 (summary index)	46.13	13.81	10.90	85.90
Mobility	66.88	22.31	2.50	100.00
Activities of daily living	54.65	24.48	4.17	95.83
Emotional well-being	47.92	21.70	4.17	100.00
Stigma	20.10	22.61	0	93.75
Social support	18.89	24.49	0	100.00
Cognitions	36.35	20.89	0	93.75
Communication	31.81	24.67	0	100.00
Bodily discomfort	45.69	20.73	0	100.00
Parkinson Fatigue Scale (PFS-16)	58.71	12.43	18.00	78.00
UPDRS-motor examination	30.05	14.06	4	58
UPDRS-complications	8.62	2.87	3	15
Zarit Burden Interview	25.30	13.47	4	64
Levodopa equivalent daily dose (mg)	1455.98	456.00	478.00	3110.00

Max, maximum; Min, minimum; PDQ-39, Parkinson's Disease Questionnaire-39 Items; SD, standard deviation; UPDRS, Unified Parkinson's Disease Rating Scale.

**Table 2:** Acceptability data of the apathy scale.

Item	Min	Max	Mean	SD	Median	Floor (%)	Ceiling (%)	Skew
1. Interested in learning?	0	3	0.92	0.98	1	45.0	6.7	0.60
2. Anything interest you?	0	2	0.45	0.62	0	61.7	0.0	1.04
3. Concern about your condition?	0	3	0.38	0.74	0	75.0	1.7	1.81
4. Much effort into things?	0	2	0.42	0.70	0	70.0	0.0	1.37
5. Looking for something to do?	0	3	0.80	1.02	0	53.3	10.0	0.98
6. Plans and goals for the future?	0	3	1.42	1.06	1	23.3	20.0	0.13
7. Do you have motivation?	0	3	1.00	1.03	1	43.3	8.3	0.48
8. Energy for daily activities?	0	3	1.33	0.86	1	20.0	5.0	−0.21
9. Someone tells you what to do?	0	3	0.62	0.96	0	65.0	6.7	1.30
10. Indifferent to things?	0	3	0.62	0.83	0	58.3	1.7	0.99
11. Unconcerned with things?	0	3	1.00	0.96	1	35.0	10.0	0.70
12. Need to be pushed?	0	3	0.87	0.98	1	46.7	8.3	0.81
13. Neither happy nor sad?	0	3	1.23	1.00	1	26.7	13.3	0.35
14. Consider yourself apathetic?	0	3	0.50	0.85	0	70.0	3.3	1.48
*Total score*	1	24	11.55	6.49	10	3.3	3.3	0.32

Max, maximum; Min, minimum; SD, standard deviation; Skew, skewness.

**Table 3:** Corrected item-total correlations.

Item	Coefficient^*∗*^
1. Interested in learning?	0.48
2. Anything interest you?	0.49
3. Concern about your condition?	0.16
4. Much effort into things?	0.22
5. Looking for something to do?	0.50
6. Plans and goals for the future?	0.44
7. Do you have motivation?	0.46
8. Energy for daily activities?	0.47
9. Someone tells you what to do?	0.31
10. Indifferent to things?	0.45
11. Unconcerned with things?	0.46
12. Need to be pushed?	0.46
13. Neither happy nor sad?	0.21
14. Consider yourself apathetic?	0.48

^*∗*^Spearman's rank correlation coefficient.

**Table 4:** Differences in assessments between baseline and follow-up^*∗*^.

Total score	Baseline				Follow-up				Wilcoxon test (*p*)
	Mean	SD	Min	Max	Mean	SD	Min	Max	
Apathy Scale	11.69	6.67	1	24	12.34	6.52	1	25	0.46
PFS-16	60.17	11.48	32	78	49.60	14.40	16	76	0.0001
UPDRS III	29.04	13.82	4	55	22.89	11.55	2	44	0.0007
UPDRS IV	8.56	2.66	3	13	4.36	2.42	0	11	<0.0001
Beck Anxiety Inventory	20.12	9.72	0	41	13.60	10.39	1	42	0.0003
Beck Depression Inv.-II	18.45	9.71	1	46	12.64	10.31	0	49	0.0017
NMSS	83.83	33.35	29	167	48.13	29.79	3	124	<0.0001
PDQ-39	46.74	13.59	10.9	85.9	33.66	16.87	4.49	72.44	<0.0001
Zarit Burden Interview	24.27	13.67	4	64	24.40	14.28	1	61	0.64

^*∗*^In 53 patients who completed the study. PFS-16, Parkinson's Fatigue Scale; Beck Depression Inv.-II, Beck Depression Inventory-II; NMSS, Non-Motor Symptoms Scale; PDQ-39, Parkinson's Disease Questionnaire-39 Items; UPDRS, Unified Parkinson's Disease Rating Scale.

## Data Availability

The data set used to support the findings of this study is available from the corresponding author upon reasonable request and after approval by the sponsor of the study (ADEQUA Study, AbbVie S.L.U.; this trial is registered with NCT02289729).
